# Antiparasitic Activity of Narciclasine and Evaluation of Its Effects on Plasma Membrane and Mitochondria of *Trypanosoma cruzi*

**DOI:** 10.1021/acsomega.4c09867

**Published:** 2025-01-14

**Authors:** Kaio S. Gomes, Thais A. Costa-Silva, Warley S. Borges, Beatriz A. Andrade, Dayana A. Ferreira, Andre G. Tempone, David Ryffel, David Sarlah, João Henrique G. Lago

**Affiliations:** † Center for Natural and Human Sciences, Federal University of ABC, 09210-180 São Paulo, SP, Brazil; ‡ Department of Chemistry, Federal University of Espírito Santo, 29075-910 Vitoria, ES, Brazil; § Physiopathology Laboratory, Butantan Institute, 05503-900 São Paulo, SP, Brazil; ∥ Roger Adams Laboratory, University of Illinois at Urbana−Champaign, 61801 Urbana, Illinois, United States; ⊥ Department of Chemistry, Wiess School of Natural Sciences, Rice University, 77005 Houston, Texas, United States

## Abstract

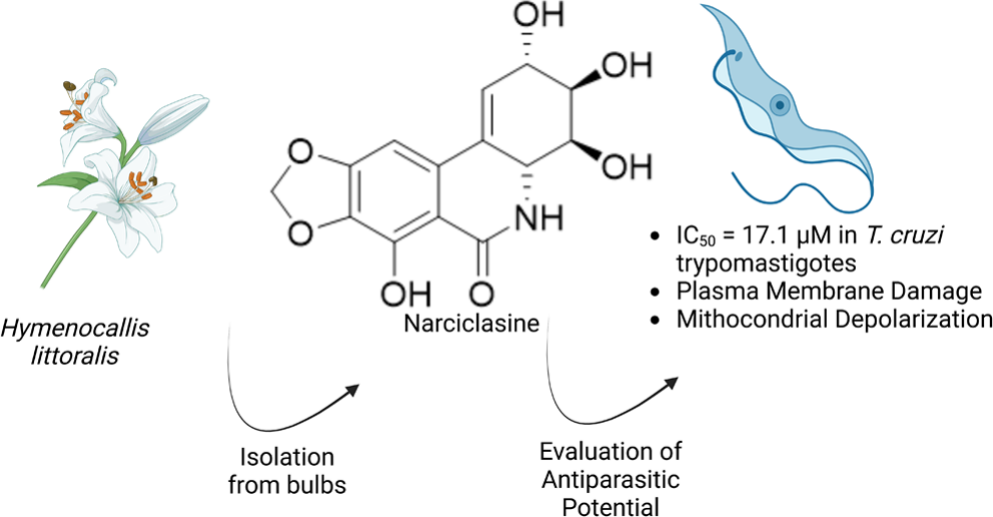

The EtOAc extract from bulbs of *Hymenocallis littoralis* (Amaryllidaceae) exhibited antiprotozoal activity against *Trypanosoma cruzi* and afforded the alkaloids narciclasine (**1**), 7-deoxynarciclasine (**2**), and narciclasine-4-O-β-D-xylopyranoside (**3**). *In silico* studies showed adequate predictions for drug-likeness for alkaloids **1** and **2**, with adherence to Lipinski′s rules of five and no alerts for PAINS. When tested against clinical forms of *T. cruzi*, alkaloid **1** displayed *in vitro* effectiveness with IC_50_ values of 17.1 μM (trypomastigotes) and 8.2 μM (amastigotes), with no mammalian cytotoxicity for NCTC cells (CC_50_ > 200 μM), similar to the standard drug benznidazole. Alkaloid **3** exhibited moderate activity against intracellular amastigotes (IC_50_ = 64.6 μM) and no activity to trypomastigotes, whereas **2** was inactive against both forms of the parasite. These results suggested that free hydroxyl groups at the C-7 and C-4 positions are involved in the potency of the alkaloids. Considering the most potent and selective compound, the lethal action of alkaloid **1** was investigated against extracellular forms (trypomastigotes). Using the fluorescent probe Sytox Green, it was observed that alkaloid **1** presented a dual effect in the plasma membrane at different concentrations from a noninterfering action (at the IC_50_) to a significant alteration in the membrane permeability (IC_90_). At all tested concentrations, alkaloid **1** induced a dose-dependent depolarization of the mitochondrial membrane potential, leading to the lethal effect on *T. cruzi*. These results suggest alkaloid **1** as a new hit compound, eliminating both clinical forms of the parasite and successful *in silico* drug-like parameters for an oral candidate for Chagas disease.

## 1 Introduction

Chagas disease (CD) is caused by the protozoan*Trypanosoma cruzi*, an endemic disease in Latin America with more than 7 million infected people.^1,2^ The infection is caused by insect vectors, allowing the access of parasites to the bloodstream; however, other transmission routes, such as blood transfusions, ingestion of contaminated food, and congenital, are also possible.^3^ The currently available drugs for CD treatment are benznidazole and nifurtimox, drugs with severe side effects and limited effectiveness according to the recommended by DND*i* (Drugs for Neglected Diseases *Initiative*).^4^

Amaryllidaceae are an important plant family in the search for alkaloids. Since the initial isolation of lycorine, more than 600 different compounds were already isolated,^5−7^ several of these displaying important bioactivities such as acetylcholinesterase inhibition,^8^ antifungal,^9^ cytotoxic,^10−15^ and antiviral.^16,17^ However, except for antimalarial potential,^18^ limited information about their action toward parasitic agents is available, especially against trypanosomatids parasites.^19^

In this study, the antiprotozoal activity of alkaloids narciclasine (**1**), 7-deoxynarciclasine (**2**), and narciclasine-4-O-β-D-xylopyranoside (**3**), isolated from bioactive EtOAc extract from bulbs of *Hymenocallis littoralis*, was evaluated *in vitro* against extra (trypomastigote) and intracellular (amastigote) forms of *T. cruzi*. Furthermore, the effects caused by alkaloid **1** on the membrane of this parasite were investigated.

## 2 Results and Discussion

### 2.1 Chemical Characterization of **1–3**

**Figure 1:**
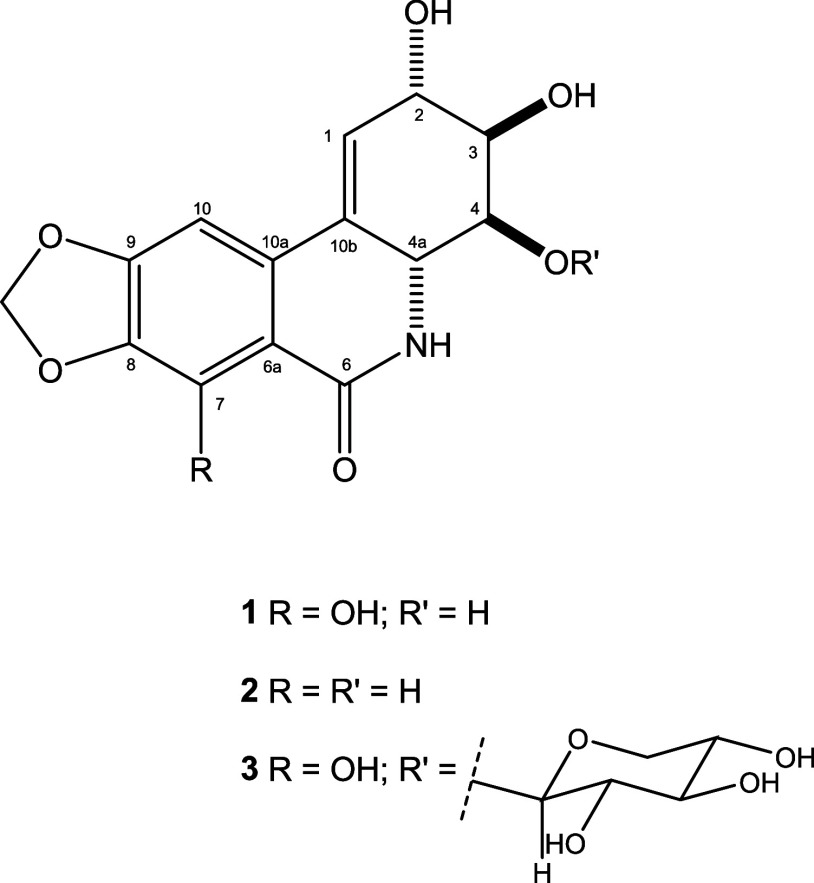
Chemical structures of alkaloids **1** - **3** isolated from bulbs of *H. littoralis*

NMR (^1^H and ^13^C) data of **1–3** were compared with those previously reported,^20−22^ allowing the characterization of narciclasine, 7-deoxynarciclasine, and narciclasine-4-O-β-D-xylopyranoside (Figure 1), respectively.

**Figure 2:**
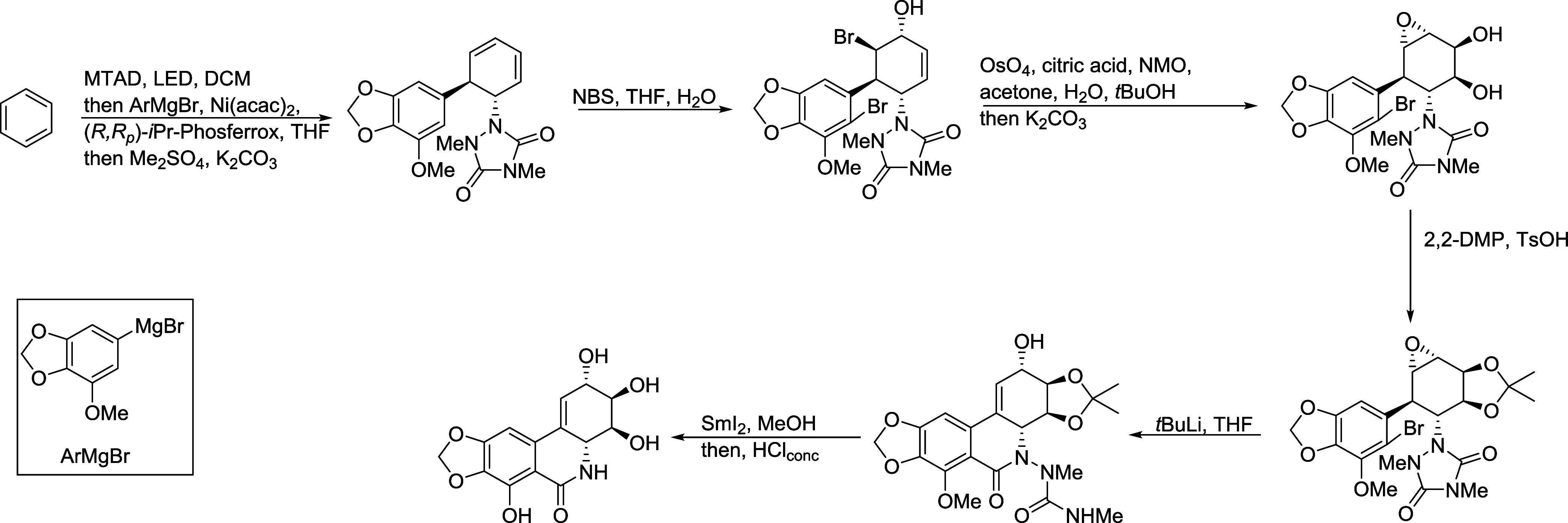
Synthesis of alkaloid **1** performed based on the Ni-catalyzed dearomative *trans*-1,2-carboamination of benzene.

Alkaloids **2** and **3** were obtained as the main metabolites from EtOAc extract from bulbs of *H. littoralis*; however, the amount of isolated **1** (0.9 mg) proved to be insufficient for the conduction of bioassays. Thus, the synthesis of this alkaloid was accomplished based on the Ni-catalyzed dearomative *trans*-1,2-carboamination of benzene,^23^ as indicated in Figure 2. Using this approach, a white solid was obtained, which displayed NMR data identical to those recorded for natural **1**.

### 2.2 In Silico Studies

**Figure 3:**
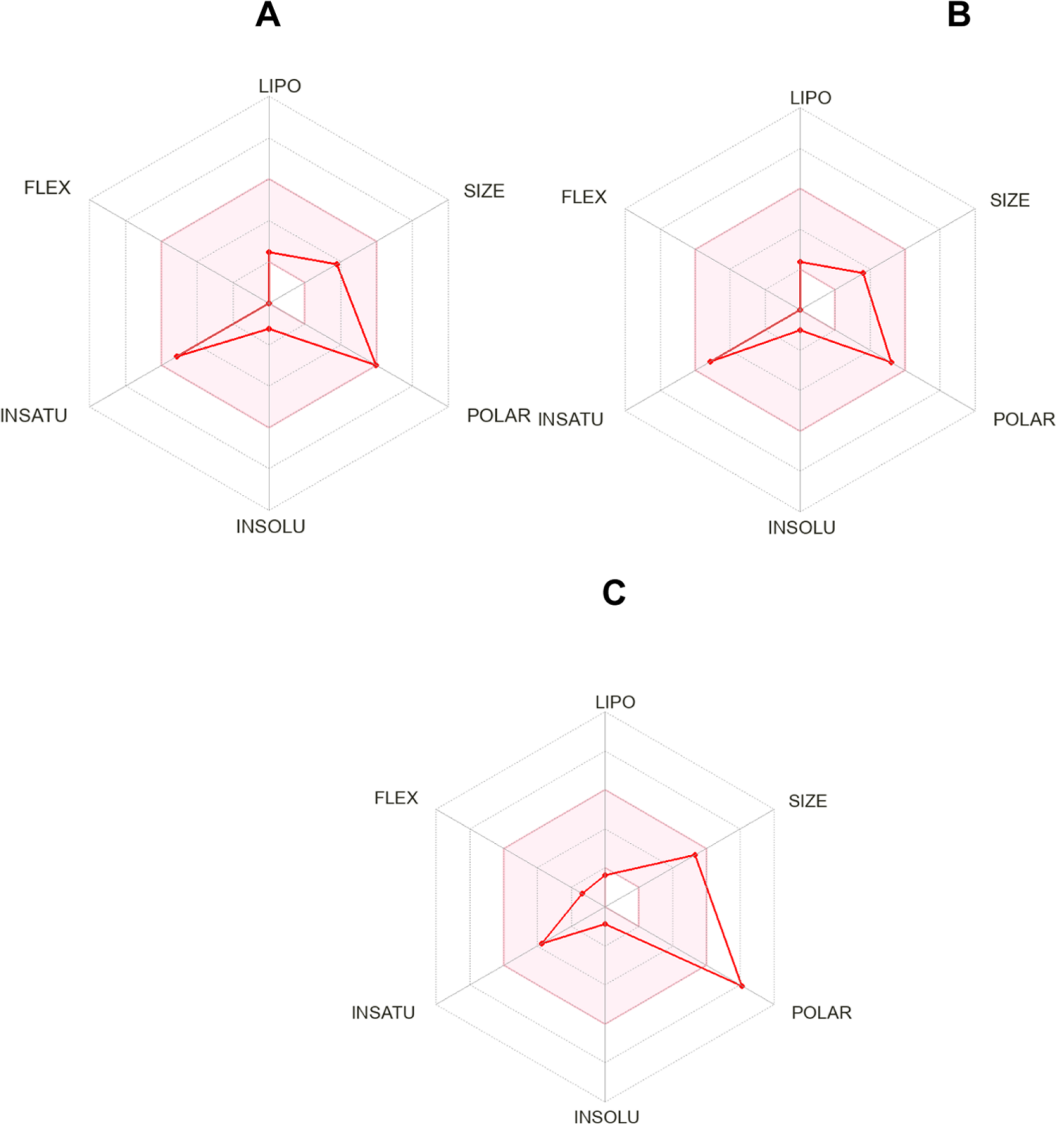
*In silico* study for drug-likeness using the SwissADME tool to **1** (A), **2** (B), and **3** (C). In this bioavailability radar, the red area represents the optimal range for each property.

To investigate different physicochemical properties, pharmacokinetic (PK) parameters, and drug-likeness of **1–3**, an *in silico* study was performed using the SwissADME tool.^24^ As observed in Figure 3, the bioavailability radar of **1–3** showed excellent adherence to the evaluated parameters, except for polarity to alkaloid **3**. Additionally, as indicated in Table 1, tested alkaloids displayed good solubility in water, with log *P*_o/w_ values of 1.47 (**1**), 1.31 (**2**), and 1.69 (**3**), considered an important parameter to deliver lead compounds.^25^ The analysis of the binding to cytochrome CYP 450 isoenzymes, responsible for the metabolization of different xenobiotics, suggested that **1–3** are not promiscuous molecules. Furthermore, no alert was evidenced for PAINS for **1–3**.^24^ However, no violation of Lipinski′s rules of five (RO5) and adhesion to different filters such as Ghose, Veber, Egan, and Muegge were observed for alkaloids **1** and **2** but not for **3**. Taking together, the obtained results suggested that **1** and **2** can act as hit compounds against *T. cruzi*.

**Table 1:** Physicochemical Properties, Pharmacokinetic, and Druggability Predictions for **1–3**

**Physicochemical Properties**	**1**	**2**	**3**
Num. heavy atoms	22	21	31
Num. arom. heavy atoms	6	6	6
Fraction Csp^3^	0.36	0.36	0.53
Num. rotatable bonds	0	0	2
Num. H-bond acceptors	7	6	11
Num. H-bond donors	5	4	7
*Lipophilicity*			
log *P*_o/w_(iLOGP)	1.47	1.31	1.69
*Pharmacokinetics*			
BBB permeant	no	no	no
P-gp substrate	no	no	no
CYP1A2 inhibitor	no	no	no
CYP2C19 inhibitor	no	no	no
CYP2C9 inhibitor	no	no	no
CYP2D6 inhibitor	no	no	no
CYP3A4 inhibitor	no	no	no
*Drug-likeness*			
Lipinski	yes	yes	no
Ghose	yes	yes	no
Veber	yes	yes	no
Egan	yes	yes	no
Muegge	yes	yes	no
Bioavailability Score	0.55	0.55	0.17
*Medicinal Chemistry*			
PAINS alert	0	0	0
Synthetic Accessibility	4.09	4.01	5.28

### 2.3 Anti-*T. cruzi* Activity

Crude EtOAc extract from bulbs of *H. littoralis* displayed activity against trypomastigotes of *T. cruzi* (100% mortality at 200 μg/mL). Therefore, this extract was subjected to bioactivity-guided fractionation, the antiparasitic effect being observed in fractions B and D (100% mortality at 200 μg/mL), after chromatographic separation over Sephadex LH-20. As evidenced by HPLC analysis, these fractions were composed of different lycorine-type alkaloids and were purified to afford **1–3**.

The *in vitro* biological activity of obtained alkaloids was assayed against the protozoan parasite *T. cruzi*, and their toxicity was evaluated against NCTC cells. As observed in Table 2, **1** exhibited potency against intracellular amastigotes of *T. cruzi* with an IC_50_ of 8.2 μM and no cytotoxicity (CC_50_ > 200 μM for NCTC cells) to afford a SI > 24.3, similar to that previously reported.^19^ Considering the activity against trypomastigotes of *T. cruzi*, **1** exhibited an IC_50_ of 17.1 μM and a SI > 11.7. These results indicated that tested **1** displayed potency comparable to that of the positive control benznidazole, which displayed IC_50_ values of 5.5 and 15.7 μM against amastigotes and trypomastigotes, respectively. On the other hand, **3** exhibited moderate activity against amastigotes (IC_50_ of 64.6 μM) with no activity against the trypomastigotes. Considering the chemical structures of these compounds, it could be suggested that the presence of xylopyranoside unity at the C-4 position in **3** might have contributed to the observed effect on the parasite. Finally, it was observed that **2** was inactive against both parasite forms. This effect might be caused by the presence of free hydroxyl groups at the C-4 and C-7 positions. Due to the effective activity against the two clinical forms of the parasite, we evaluated the effect of the most potent and selective compound **1** in the plasma membrane (SYTOX Green probe) and mitochondria using the probes rhodamine (Rd123) and the live–dead fluorescent probe, propidium iodide (PI).

**Table 2:** Anti-*T. cruzi* Activities (Trypomastigotes and Amastigotes), Cytotoxicity in Mammalian Cells (NCTC), SI of **1–3**, and Positive Control Benznidazole^a^^,^^b^^,^^c^^,^^d^

	**IC**_**50**_**(μM)**		**CC**_**50**_**(μM)**	**SI**	
**Compound**	Trypomastigote	Amastigote	NCTC	Trypomastigote	Amastigote
**1**	17.1 ± 2.8	8.2 ± 0.3	>200	>11.7	>24.3
**2**	NA	NA	>200		
**3**	NA	64.6 ± 0.7	>200		>3.1
**Benznidazole**	15.7 ± 0.9	5.5 ± 0.8	>200	>12.7	>36.4

a IC_50_: 50% inhibitory concentration (trypomastigote and amastigote forms of *T. cruzi*).

b CC_50_: 50% cytotoxic concentration (NCTC cells).

c NA: nonactive.

d SI: selectivity index (CC_50_/IC_50_).

### 2.4 Evaluation of Alterations in Plasma Membrane Permeabilization Using SYTOX Green Dye

**Figure 4:**
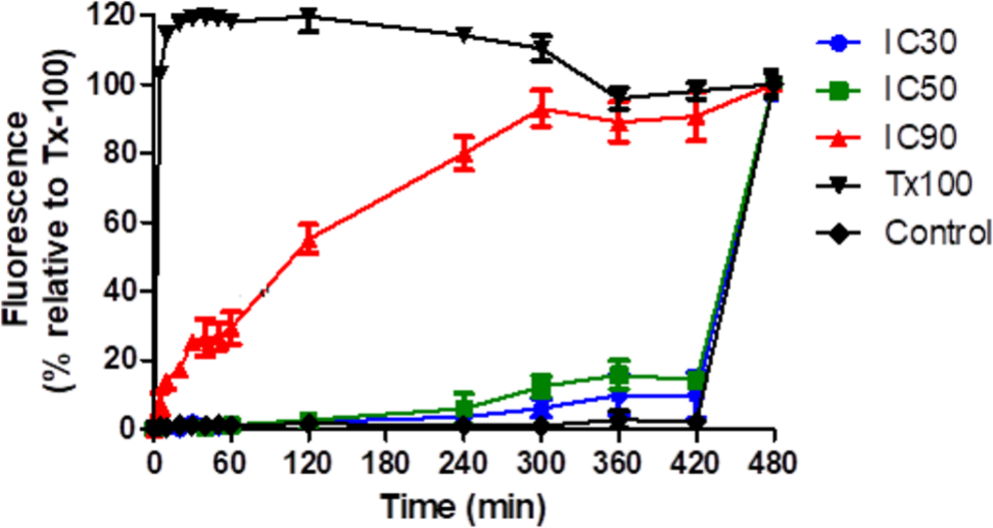
Plasma membrane permeability of **1** on trypomastigotes of *T. cruzi*. The entrance of SYTOX Green dye was monitored fluorometrically for up to 420 min. Untreated trypomastigotes and treated with Tx-100 were used to achieve minimal and maximal permeabilization, respectively. IC_30_, IC_50_, and IC_90_ values correspond to 6, 17, and 67 μM, respectively.

The effect of **1** on the plasma membrane permeabilization was investigated using the fluorescence dye SYTOX Green. As a result, **1** showed a dual effect in the plasma membrane of trypomastigotes at the three different tested concentrations, from a noninterfering action (at the IC_50_) to a significant alteration in the membrane permeability at the IC_90_. An expressive increase in the fluorescence levels was observed to **1** at 20 min incubation at the IC_90_ when compared to untreated parasites. After 480 min (8 h) of incubation of **1** at IC_90_, the fluorescence was quite similar to that observed in the parasite treated only with Triton X-100 at 0.5% (Figure 4). To produce the observed fluorescence caused by the binds of SYTOX green dye to intracellular nucleic acids, membrane permeabilization is necessary.^26^ At smaller concentrations (IC_30_ and IC_50_), **1** showed no significant interference in the membrane. Compounds such as Triclosan, a bacteriostatic agent used as a preservative in cosmetics and medicines, have been described in the literature with similar effects.^27^ Thus, we suggest that compound **1** might also have a dose-dependent mechanism of action in *T. cruzi*.

### 2.5 Evaluation of Alterations in the Mitochondrial Membrane Potential Caused by **1** Using SYTOX Green Dye

**Figure 5:**
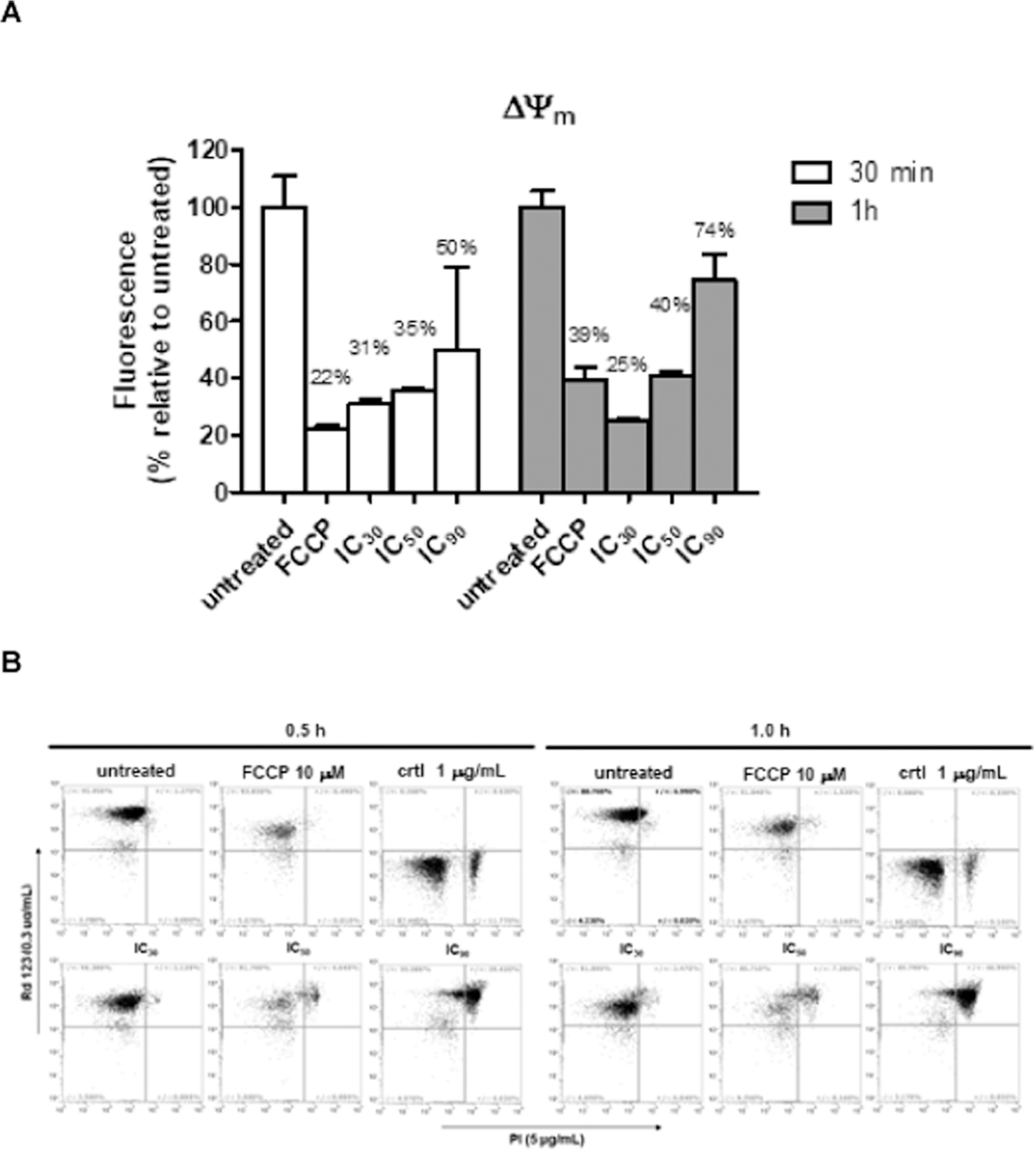
Alteration of mitochondrial membrane potential of **1** in trypomastigotes of *T. cruzi*. (A) Changes of Rd123 fluorescence determined by flow cytometry and reported as a percentage relative to untreated parasites. Maximal and minimal fluorescence of Rd123 were achieved by nontreatment and treatment with FCCP (10 μM), respectively. IC_30_, IC_50_, and IC_90_ values correspond to 6, 17, and 67 μM, respectively. (B) Flow cytometry graphs showing in *y*-axis Rd123 fluorescence and *x*-axis PI fluorescence.

The effect of **1** on the mitochondrial membrane potential was investigated using a combination of rhodamine 123 (Rd123) and propidium iodide (PI) by flow cytometry. As a result, the Rd123 fluorescence decreased in the parasites treated with **1** after 60 min in comparison to that of the untreated trypomastigotes (Figure 5A). Furthermore, an enhancement of PI fluorescence levels was observed (Figure 5B), confirming the effect of plasma membrane permeabilization determined by SYTOX Green. Otherwise, nonpermeabilized parasites incubated with **1** (positive to Rd123 and negative to PI) displayed a reduced accumulation of Rd123, an analogous effect observed to the parasites treated with FCCP at 10 μM. Thus, the bioactive alkaloid **1** effect simultaneously affects the mitochondrial membrane potential and the plasma membrane permeability. These effects on trypomastigotes forms of *T. cruzi* were previously reported for other natural alkaloids.^28,29^ Furthermore, mitochondrial dysfunction was observed in different natural products with anti-*T. cruzi* activity.^30,31^ In the present study, it was observed that the permeabilization of the plasma membrane plays an important role in the effect of **1** against trypomastigote forms of *T. cruzi*. As reported in the literature,^32^ the lipid and protein compositions of membranes from *T. cruzi* and mammalian cells are quite different, which can, at least in part, explain the reduced cytotoxicity of **1** to NCTC cells (Table 2). At all tested concentrations, a dose-dependent depolarization of the mitochondrial membrane potential induced by **1** was observed, leading to the lethal effect on *T. cruzi*.

## 3 Conclusions

The present work demonstrates that alkaloid narciclasine (**1**) exhibited promising potential against *T. cruzi* parasites, especially against intracellular forms (amastigotes), with reduced toxicity against NCTC cells. On the other hand, structurally related alkaloids 7-deoxynarciclasine (**2**) and narciclasine-4-O-β-D-xylopyranoside (**3**) exhibited no activity against trypomastigotes. Concerning amastigotes, only **3** showed a moderate potential. Considering the selectivity of **1** to the trypomastigotes, it was suggested that the lethal action in the parasite might be dose-dependent and affect the plasma membrane or the mitochondria. Based on the obtained biological results and the possibility of accessing higher amounts of these alkaloids using synthetic approaches, narciclasine (**1**) could be used as a prototype in drug design studies for the development of new drugs against *T. cruzi*.

## 4 Material and Methods

### 4.1 General

NMR spectra were recorded on a Varian INOVA spectrometer, operating at 500 MHz for ^1^H and 125 MHz for ^13^C nuclei, using CD_3_OD or DMSO-*d*_6_, both from Sigma-Aldrich, as the solvent and internal standards. Chemical shifts are reported in δ units (ppm) and coupling constants (*J*) in Hz. Silica gel 60 (Merck) and Sephadex LH-20 (GE Healthcare) were used for column chromatography, while silica gel F_254_ (Macherey-Nagel, MN) was used for analytical TLC. HPLC analyses were conducted on an UltiMate 3000 BioRS System from Thermofisher Scientific equipped with a UVD-170OU diode array detector and a Phenomenex Kinetex EVO C_18_ (5 μm, 150 mm × 4.6 mm, flow rate: 1.0 mL/min) column. Other reagents and solvents were used without further purification.

### 4.2 Plant Material

Bulbs of *H. littoralis* (Jacq.) Salisb. were collected in São Félix, Bahia, Brazil, in March 2022. A specimen voucher was compared to that deposited in the Herbarium of *Jardim Botânico do Rio de Janeiro* (Rio de Janeiro, Brazil) and received a registration code at SisGen A4123E4.

### 4.3 Extraction and Isolation

Dried bulbs (90 mg) were exhaustively extracted using EtOAc to afford 37 mg of the crude extract. Part of this material (35 mg) was subjected to column chromatography over Sephadex LH-20 (MeOH as eluent) to afford five groups (A – E). Bioactive group D (5.8 mg) was purified by semiprep. HPLC to give **1** (0.9 mg) and **2** (3.4 mg), whereas bioactive fraction B (13.4 mg) was shown to be composed of **3**.

#### 4.3.1 Narciclasine (**1**)

White amorphous solid (100% of purity by HPLC). ^1^H NMR (DMSO-*d*_6_, 500 MHz): δ 6.54 (s, H-10), 6.13–6.12 (m, H-1), 5.90 (s, OCH_2_O), 4.70 (dq, *J* = 7.0 and 1.0 Hz, H-4a), 4.12–4.08 (m, H-4), 3.99–3.95 (m, H-2), 3.74–3.71 (m, H-3). ^13^C NMR (DMSO-*d*_6_, 125 MHz): δ 171.0 (C-6), 152.6 (C-9), 143.8 (C-7), 137.2 (C-8), 133.5 (C-10a), 131.9 (C-10b), 125.3 (C-6a), 124.8 (C-1), 101.8 (OCH_2_O), 93.2 (C-10), 71.7 (C-2), 70.5 (C-3), 70.4 (C-4), 55.1 (C-4a).

#### 4.3.2 7-Deoxynarciclasine (**2**)

White amorphous solid (100% purity by HPLC). ^1^H NMR (DMSO-*d*_6_, 500 MHz): δ 7.30 (s, H-7), 6.85 (s, H-10), 5.97 (dt, *J* = 7.2 and 1.8 Hz, H-1), 5.85 (s, OCH_2_O), 4.51 (dq, *J* = 7.0 and 1.0 Hz, H-4a), 3.99–3.95 (m, H-4), 3.85–3.81 (m, H-2), 3.61–3.58 (m, H-3). ^13^C NMR (DMSO-*d*_6_, 125 MHz): δ 164.0 (C-6), 149.0 (C-9), 147.5 (C-8), 133.4 (C-10b), 130.0 (C 10a), 123.6 (C-6a), 122.7 (C-1), 106.8 (C-7), 103.9 (C-10), 101.8 (OCH_2_O), 72.5 (C-2), 70.5 (C-3), 70.0 (C-4), 53.8 (C-4a).

#### 4.3.3 Narciclasine-4-O-β-D-xylopyranoside (**3**)

White amorphous solid (99% purity by HPLC). ^1^H NMR (CD_3_OD, 500 MHz): δ 6.80 (s, H-10), 6.22 (ddd, *J* = 4.8, 2.5, and 1.0 Hz, H-1), 6.02 (br s, OCH_2_O), 4.44 (ddd, *J* = 8.9, 2.5, and 1.2 Hz, H-4a), 4.41 (d, *J* = 7.5 Hz, H-1’), 4.30 (ddd, *J* = 4.8, 2.5, and 1.2 Hz, H-2), 4.07 (m, H-3), 4.05 (m, H-5′a), 3.30 (m, H-5′b), 4.02 (dd, *J* = 8.9 and 2.2 Hz, H-4), 3.61 (ddd, *J* = 10.4, 8.8, and 5.4 Hz, H-4’), 3.40 (t, *J* = 8.8 Hz, H-3′), 3.35 (m, H-2′). ^13^C NMR (CD_3_OD, 125 MHz): δ 170.4 (C-6), 154.5 (C-9), 146.6 (C-7), 135.8 (C-8), 133.2 (C-10a), 132.1 (C-10b), 123.8 (C-1), 107.1 (C-6a), 104.0 (C-1’), 103.8 (OCH_2_O), 97.4 (C-10), 79.6 (C-4), 77.6 (C-3′), 74.8 (C-2’), 72.0 (C-3), 71.1 (C-4’), 70.3 (C-2), 67.3 (C-5′), 52.1 (C-4a).

### 4.4 Synthesis of Narciclasine (**1**)

Synthesis of narciclasine (**1**) was based on the Ni-catalyzed dearomative *trans*-1,2-carboamination of benzene, as previously described.^23^ Using this approach, 90 mg of **1** was obtained (26% overall yield and 100% of purity by HPLC).

### 4.5 In silico Studies and Drug-likeness Assessment

Prediction of physicochemical properties and pharmacokinetic parameters of **1–3** were determined *in silico* using the platform SwissADME (http://www.swissadme.ch/).^24^ These computational tool analysis diverse parameters were such as (i) physicochemical properties (number of rotatable bonds, number of H-bond donors and H-bond acceptors), (ii) lipophilicity (log P value), (iii) pharmacokinetics (BBB permeant, P-gp substrate, and CYP 450 inhibitors), (iv) drug-likeness with Big-Pharma filters including Lipinski (Pfizer), Veber (GlaxoSmithKline), Muegge (Bayer), (v) alert for pan-assay interference compounds (PAINS), and (vi) synthetic accessibility.

### 4.6 Animal Models, Mammalian Cells, and Parasite Maintenance

Information regarding animal models, mammalian cells, and parasite maintenance has previously been reported.^33,34^ Performed procedures were approved by the Animal Care and Use Committee from the *Instituto Adolfo Lutz* - Secretary of Health of São Paulo State (Project CTC 72-J/2017).

### 4.7 Evaluation of Activity Against Trypomastigote and Amastigote Forms of *T. cruzi*

Activity against trypomastigote and amastigote forms of *T. cruzi* of **1–3** was evaluated by means of determination of IC_50_ values, as previously reported.^35^

### 4.8 Evaluation of Cytotoxicity Against Mammalian (NCTC) Cells

Toxicity against mammalian (NCTC) cells of **1–3** was evaluated by means of determination of CC_50_ values, as previously described.^36,37^ Selectivity index values were determined by dividing CC_50_ (against NCTC cells) by IC_50_ (against trypomastigotes and amastigotes) values.

### 4.9 SYTOX Green Assay for Cell Membrane Permeabilization

Assays for the determination of cell membrane permeabilization of *T. cruzi* were performed using SYTOX Green dye, as previously reported.^38^ Briefly, alkaloid **1** was added (*t* = 0 min) at IC_30_ (6 μM), IC_50_ (17 μM), and IC_90_ (67 μM), and fluorescence was measured every 1 h for up to 8 h using a fluorimetric microplate reader (FilterMax F5Multi-Mode Microplate Reader-Molecular Devices). The maximum permeabilization was obtained with 0.5% Triton X-100.

### 4.10 Flow Cytometry Analysis for Mitochondrial Membrane Potential (ΔΨ*m*)

Analysis of alteration on mitochondrial membrane potential (ΔΨm) in *T. cruzi* was performed with rhodamine 123 (Rd123) and propidium iodate (PI), as previously reported.^26,39^ Briefly, trypomastigotes were treated with **1** at IC_30_ (6 μM), IC_50_ (17 μM), and IC_90_ (67 μM) in HBSS + Glu for 30 min and 1 h of incubation at 26 °C using an Attune NxT Acoustic Focusing Cytometer (ThermoFisher). Untreated and treated trypomastigotes (FCCP 10 μM) were used as negative and positive controls, respectively.

### 4.11 Statistical Analysis

IC_30_, IC_50_, IC_90_, and CC_50_ values were calculated by using Sigmoidal dose–response curves using Graph Pad Prism 5.0 software. One-way ANOVA of variance with Tukey’s multiple comparison test was used for the significance test (*P* value). All assays were repeated at least twice.

## Supporting Information Available

Figure S1: ^1^H NMR spectrum of compound 1 (δ, DMSO-*d*_6_, 500 MHz); Figure S2: ^13^C NMR spectrum of compound 1 (δ, DMSO-*d*_6_, 125 MHz); Figure S3: ^1^H NMR spectrum of compound 2 (δ, DMSO-*d*_6_, 500 MHz); Figure S4: ^13^C NMR spectrum of compound 2 (δ, DMSO-*d*_6_, 125 MHz); Figure S5: ^1^H NMR spectrum of compound 3 (δ, CD_3_OD, 500 MHz); and Figure S6: ^13^C NMR spectrum of compound 3 (δ, CD_3_OD, 125 MHz) (PDF)
