# Supplementary material for: Antiparasitic Activity of Narciclasine and Evaluation of Its Effects on Plasma Membrane and Mitochondria of Trypanosoma cruzi
Source: ACS Omega. 2025 Jan 14;10(3):3025–32. doi: 10.1021/acsomega.4c09867 (PMC11780464; doi:10.1021/acsomega.4c09867)
Supplement: Supplementary file 1 — ao4c09867_si_001.pdf [file ao4c09867_si_001.pdf]

SUPPLEMENTARY INFORMATION

Antiparasitic activity of narciclasine and evaluation of its effects on plasma membrane and mitochondria of *Trypanosoma cruzi*

Kaio S. Gomes[a], Thais A. Costa-Silva[a], Warley S. Borges[b], Beatriz A. Andrade[c], Dayana A. Ferreira[c], Andre G. Tempone[c], David Ryffel[d], David Sarlah[e,*] João Henrique G. Lago[a,*]

[a]Center for Natural and Human Sciences, Federal University of ABC, 09210-180 São Paulo, SP, Brazil.

[b]Department of Chemistry, Federal University of Espírito Santo, 29075-910, Vitoria, ES, Brazil.

[c]Physiopathology Laboratory, Butantan Institute, 05503-900, São Paulo, SP, Brazil.

[d]Roger Adams Laboratory, University of Illinois at Urbana-Champaign, 61801, Urbana, IL, USA.

[e]Department of Chemistry, Wiess School of Natural Sciences, Rice University, 77005, Houston, TX, USA

*Corresponding authors.

David Sarlah, sarlah@illinois.edu

Department of Chemistry, Wiess School of Natural Sciences, Rice University, 77005, Houston, TX, USA

João Henrique G. Lago, joao.lago@ufabc.edu.br

Federal University of ABC, Av. dos Estados, 5001, 09210-580, Santo André, SP, Brazil

CONTENT TABLE

Figure S1 - ^{1}H NMR spectrum of compound **1** (δ,DMSO-d_6, 500 MHz) 4

Figure S2 - ^{13}C NMR spectrum of compound **1** (δ,DMSO-d_6, 125 MHz) 5

Figure S3 - ^{1}H NMR spectrum of compound **2** (δ,DMSO-d_6, 500 MHz) 7

Figure S4 - ^{13}C NMR spectrum of compound **2** (δ,DMSO-d_6, 125 MHz) 8

Figure S5 - ^{1}H NMR spectrum of compound **3** (δ,CD_3OD, 500 MHz) 10

Figure S6 - ^{13}C NMR spectrum of compound **3** (δ, CD_3OD, 125 MHz) 11

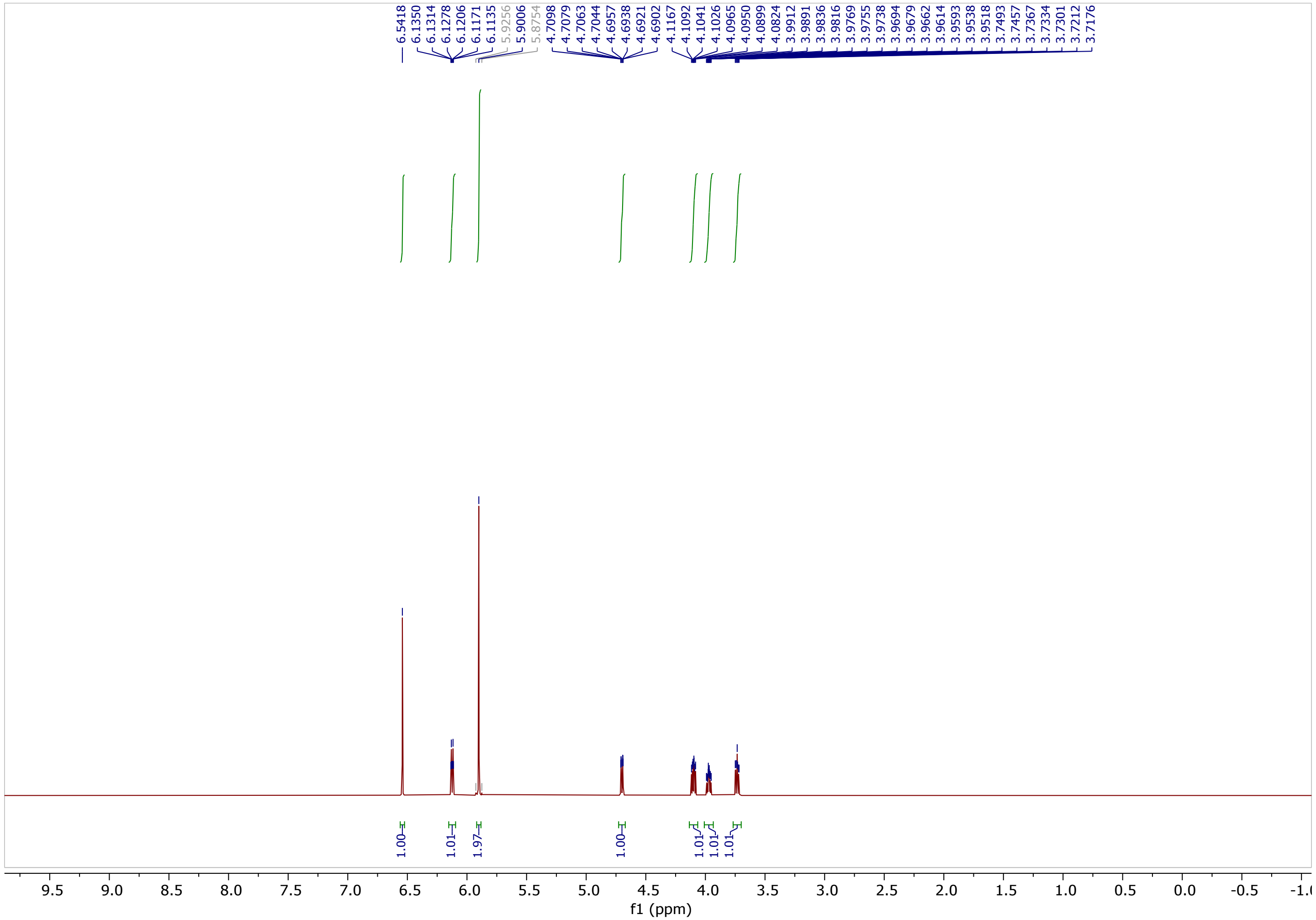

Figure S1 - ^{1}H NMR spectrum of compound **1** (δ,DMSO-d_6, 500 MHz)

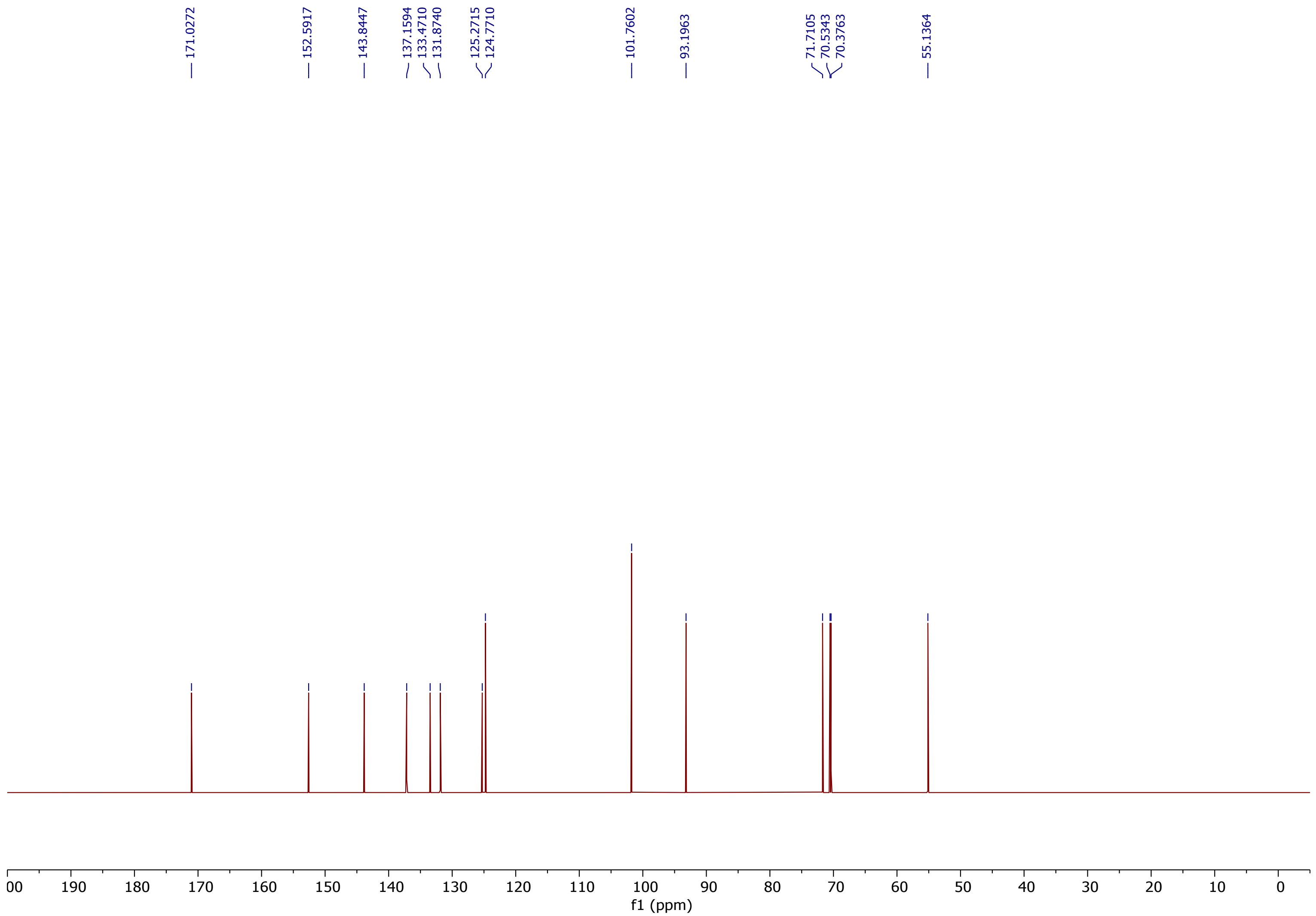

Figure S2 – ^{13}C NMR spectrum of compound **1** (δ,DMSO-d_6, 125 MHz)

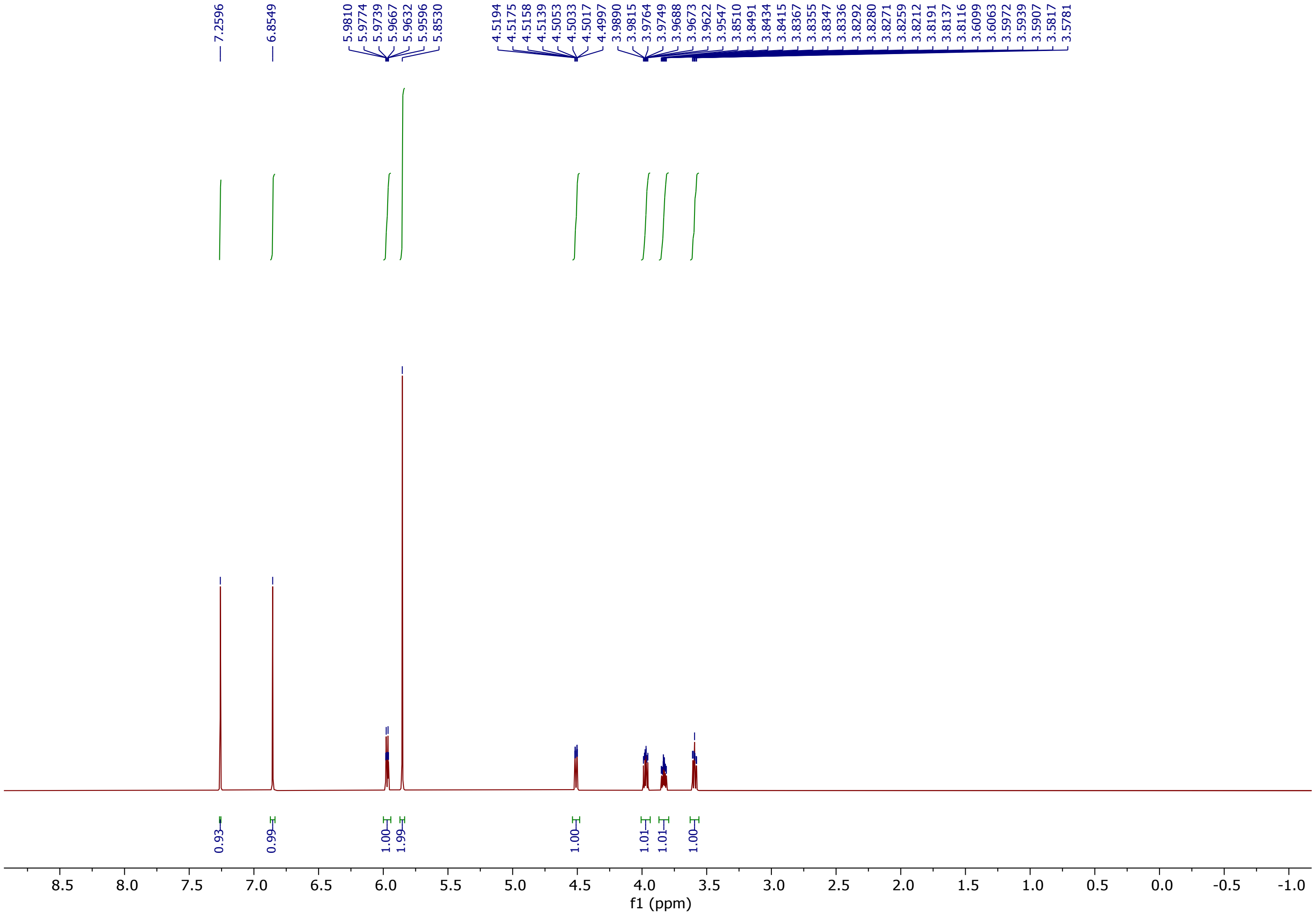

Figure S3 - ^{1}H NMR spectrum of compound **2** (δ,DMSO-d_6, 500 MHz)

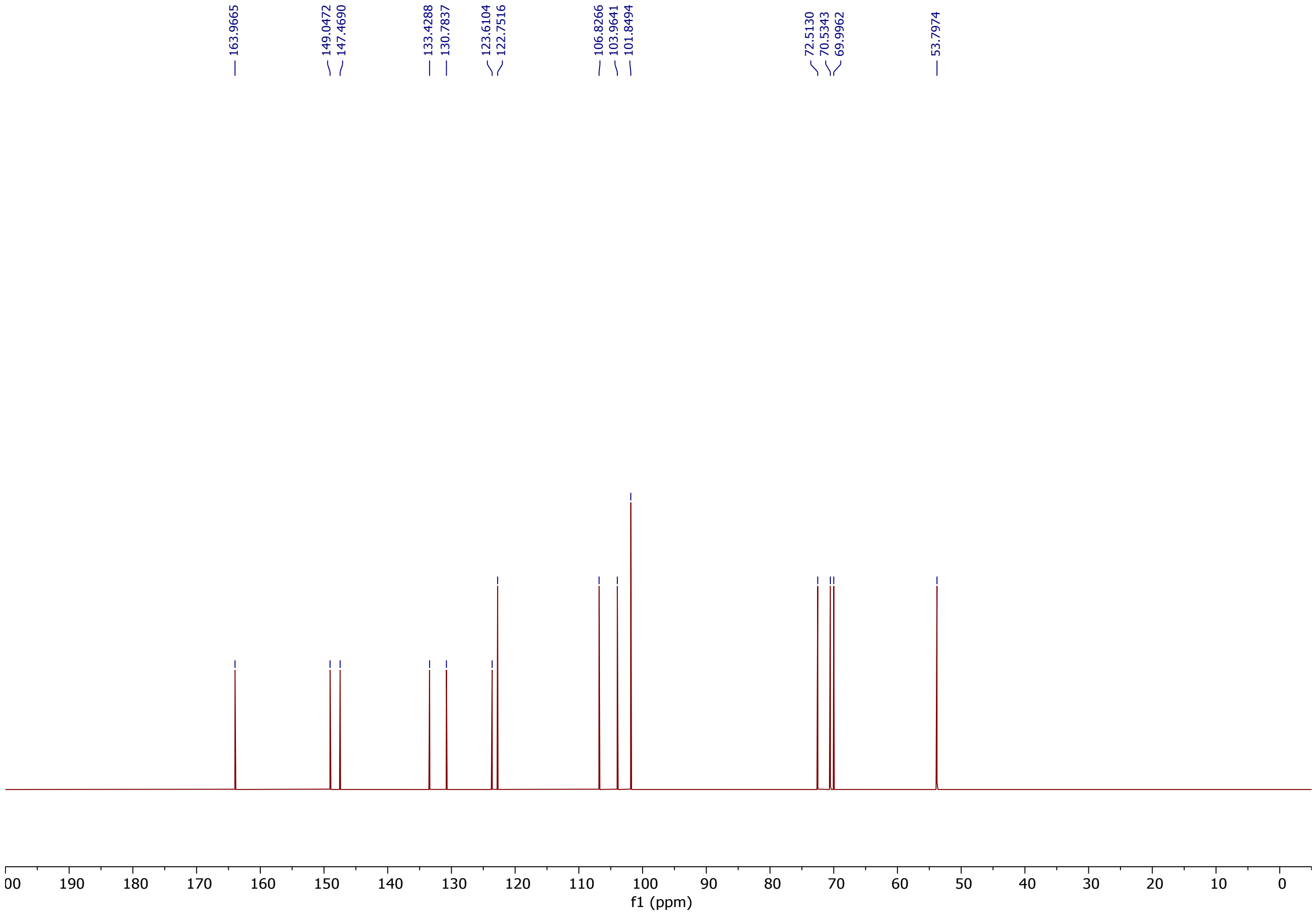

Figure S4 – ^{13}C NMR spectrum of compound **2** (δ,DMSO-d_6, 125 MHz)

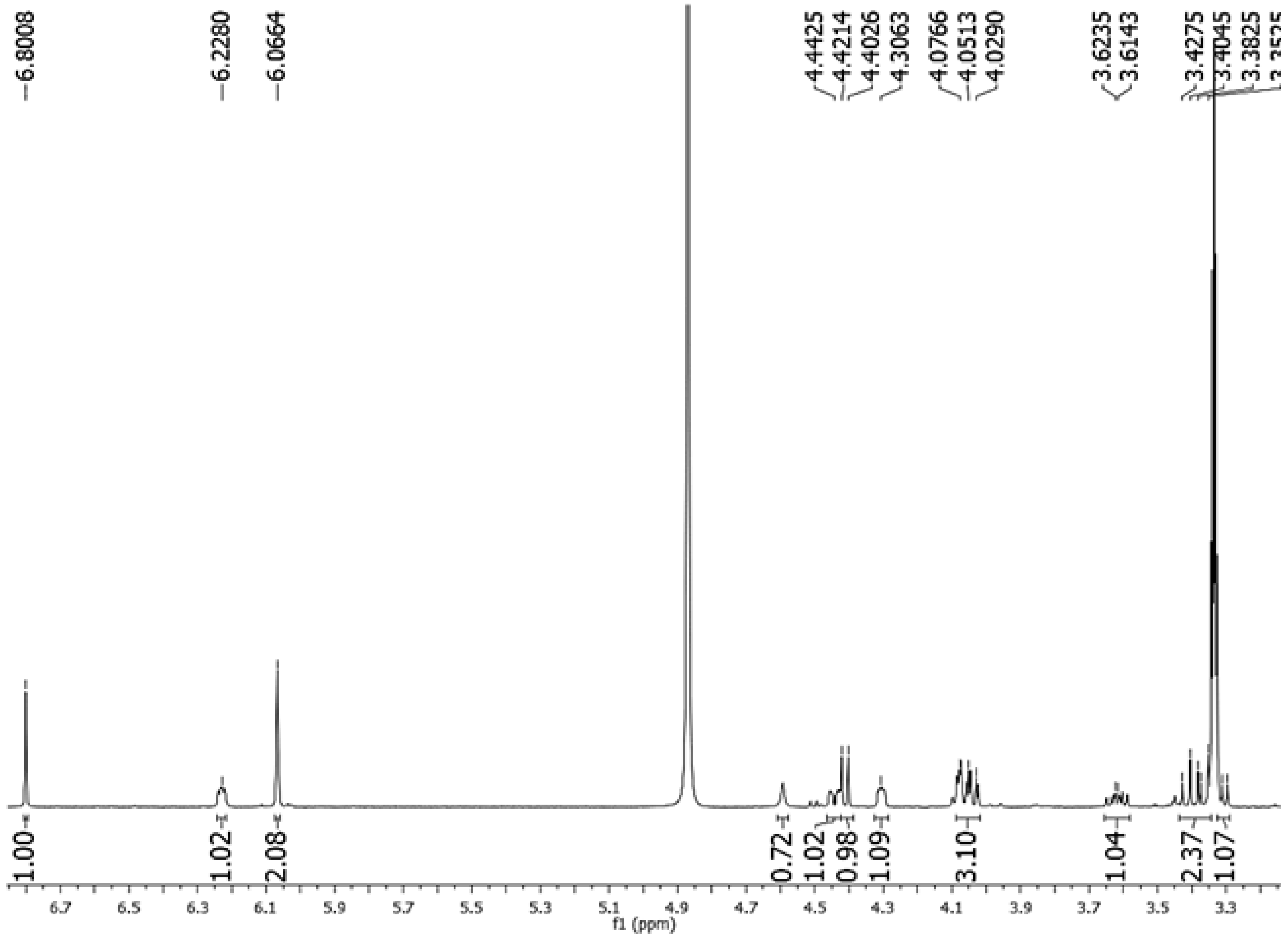

Figure S5 – ^{1}H NMR spectrum of compound **3** (δ, CD_3OD, 500 MHz)

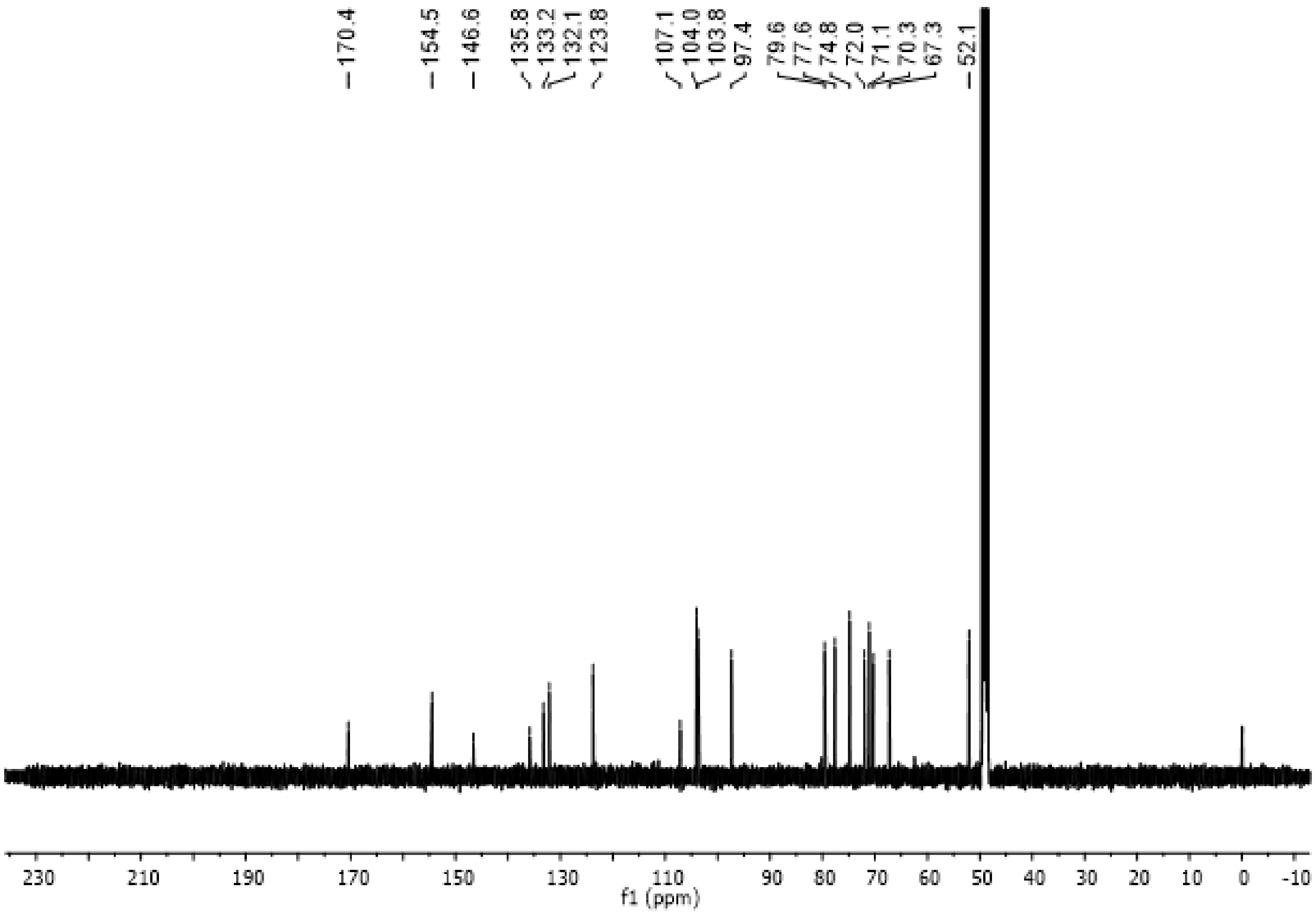

Figure S6 – ^{13}C NMR spectrum of compound **3** (δ, CD_3OD, 125 MHz)
